# Consumption of chilies and sweet peppers is associated with lower risk of sarcopenia in older adults

**DOI:** 10.18632/aging.104168

**Published:** 2021-03-26

**Authors:** Xuena Wang, Xiaohui Wu, Ge Meng, Shanshan Bian, Qing Zhang, Li Liu, Hongmei Wu, Yeqing Gu, Shunming Zhang, Yawen Wang, Tingjing Zhang, Xingqi Cao, Huiping Li, Yunyun Liu, Xiaoyue Li, Kun Song, Kaijun Niu

**Affiliations:** 1 Nutritional Epidemiology Institute and School of Public Health, Tianjin Medical University, Tianjin, China; 2 College of Pharmacy, Tianjin Medical University, Tianjin, China; 3 Department of Toxicology and Sanitary Chemistry, School of Public Health, Tianjin Medical University, Tianjin, China; 4 Department of Nutrition, The Second Hospital of Tianjin Medical University, Tianjin, China; 5 Health Management Centre, Tianjin Medical University General Hospital, Tianjin, China; 6 Institute of Radiation Medicine, Chinese Academy of Medical Sciences and Peking Union Medical College, Tianjin, China; 7 Tianjin Key Laboratory of Environment, Nutrition and Public Health, Tianjin, China; 8 Center for International Collaborative Research on Environment, Nutrition and Public Health, Tianjin, China

**Keywords:** chili, sweet pepper, sarcopenia, capsaicin, capsiate

## Abstract

Background: Sarcopenia is an aging-related loss of muscle mass and function, which induces numerous adverse outcomes. Capsaicin and capsiate, separately extracted from chilies and sweet peppers, have the potential to induce muscle hypertrophy via activation of transient receptor potential vanilloid 1. The present study aimed to investigate whether chili and sweet pepper consumption are related to sarcopenia in the elderly general population.

Methods: A cross-sectional study with 2,451 participants was performed. Dietary chili and sweet pepper consumption were assessed using a validated self-administered food frequency questionnaire. Sarcopenia was defined according to the consensus of the Asian Working Group for Sarcopenia. Logistic regressions were performed to measure the effect of chili and sweet pepper consumption on sarcopenia.

Results: The prevalence of sarcopenia was 16.1%. After adjustment for potential confounding variables, the odds ratios (95% confidence intervals) for sarcopenia across chili and sweet pepper consumption categories were 1.00 (reference) for almost never, 0.73 (0.55, 0.97) and 0.73 (0.56, 0.96) for ≤1 time/week, 0.60 (0.39, 0.90) and 0.66 (0.45, 0.95) for ≥2-3 times/week (both *P* for trend <0.01), respectively.

Conclusion: The present study showed that higher consumption of chilies and sweet peppers was related to a lower risk of sarcopenia in older adults.

## INTRODUCTION

Sarcopenia, a geriatric syndrome, is defined by an involuntary loss of muscle mass combined with a decrease in muscle strength and/or physical performance with advancing age [1]. Sarcopenia has been reported to correlate with various adverse health outcomes such as physical limitations, disability, hospitalization, and mortality [2–5]. With a rapidly expanding demographic of older people, sarcopenia leads to substantial social and economic costs and has emerged as a significant public health issue.

Peppers, including chili peppers and sweet peppers, have been reported to have antioxidant and anti-inflammatory effects, stimulate lipid metabolism, increase energy expenditure, and control diabetes [6–9]. The potential effects of peppers on muscle have drawn the attention of researchers. Scientific evidence suggests that capsaicin, the major pungent compound in chilies, can activate the transient receptor potential vanilloid 1 (TRPV1) [10]. Capsiate, a nonpungent capsaicin analog extracted from sweet peppers, is also an agonist of TRPV1 [11]. Previous studies have illustrated that TRPV1 was expressed in skeletal muscle tissues [12]. The activation of TRPV1 can induce an increased cytosolic calcium concentration that subsequently triggers the mammalian target of rapamycin (mTOR) [13]. The upregulation of the mTOR signaling pathway may result in muscle hypertrophy via increased protein synthesis [14]. Therefore, there may be potential benefits of chili and sweet pepper intake on muscle strength and physical function.

Many animal experiments have explored the detailed mechanism of capsaicin and capsiate on skeletal muscle [15, 16]. It is widely understood that findings from preclinical studies, such as animal model studies, cannot be directly applied to humans. However, the findings from previous animal experiments have not been investigated in human studies. In China, chili is among the most popular spicy foods consumed, while sweet pepper is also frequently used in daily diets [17]. From a public health perspective, we expect daily chili and sweet pepper intake to be beneficial for prevention of sarcopenia. Therefore, the purpose of our study was to investigate the cross-sectional relationship between chili and sweet pepper consumption and sarcopenia in the older population.

## RESULTS

In the present study, the prevalence of sarcopenia was 16.1% (394/2,451). Baseline characteristics of study participants according to sarcopenia status are shown in Table 1. Compared with participants without sarcopenia, those with sarcopenia tended to be older (*P* values < 0.0001), more likely to be females (*P* values < 0.0001), to have lower BMI (*P* values < 0.0001) and to have a lower proportion of high educational level (*P* values = 0.01). The "fruits and sweets'' dietary pattern score (*P* values = 0.046) and "animal foods'' dietary pattern score (*P* values < 0.001) were lower in participants with sarcopenia. The individuals with sarcopenia were more likely to be ex-smokers (*P* values < 0.01) and non-drinker (*P* values = 0.02) and to have a history of hyperlipidemia (*P* values < 0.01), diabetes (*P* values = 0.04) and stroke (*P* values < 0.0001).

**Table 1:** Age and sex-adjusted participant characteristics by sarcopenia status (n = 2,451).

**Characteristics**	**Without sarcopenia**	**With sarcopenia**	***P*^a^**
No. of subjects	2,057	394	-
Age (y)	67.3 (67.1, 67.5) ^b^	70.5 (70.0, 71.0)	<0.0001
Sex (males, %)	40.4	33.8	<0.0001
BMI (kg/m2)	25.3 (25.2, 25.5)	23.8 (23.5, 24.2)	<0.0001
Total energy intake (kcal/day)	2,089.2 (2,060.4, 2,118.0)	2,027.7 (1,960.8, 2,094.5)	0.10
"Fruits and sweets'' dietary pattern score	0.04 (0.00, 0.10)	-0.07 (-0.17, 0.00)	0.046
"Healthy'' dietary pattern score	0.00 (-0.04, 0.00)	0.04 (-0.06, 0.10)	0.49
"Animal foods'' dietary pattern score	0.11 (0.07, 0.20)	-0.08 (-0.18, 0.00)	<0.001
Physical activity (Mets × hour/week)	26.6 (25.5, 27.6)	22.5 (20.1, 25.0)	<0.01
Smoking status (%)			
Smoker	32.5	35.0	0.09
Ex-smoker	51.2	60.2	<0.01
Non-smoker	8.99	9.14	0.73
Drinking status (%)			
Everyday	13.4	11.2	0.62
Sometime	7.24	6.85	0.31
Ex-drinker	1.70	1.02	0.78
Non-drinker	72.5	80.5	0.02
Educational level (≥college grade, %)	5.74	1.27	0.01
Managers (%)	17.4	16.0	0.42
Marital status (married, %)	98.2	98.2	0.98
Household income (≥10,000 Yuan, %)	11.9	6.85	0.06
Depressive symptoms (>45, %)	9.19	12.7	0.14
Individual history of diseases (%)			
Hypertension	52.6	58.9	0.36
Hyperlipidemia	42.3	53.3	<0.01
Diabetes	12.5	17.0	0.04
Cardiovascular disease	11.3	14.2	0.44
Stroke	4.23	8.63	<0.0001
Cancer	0.58	0.25	0.44
Assessment of sarcopenia			
RASM (kg/m2)	6.37 (6.34, 6.40)	5.66 (5.59, 5.72)	<0.0001
Grip strength (kg)	24.9 (24.7, 25.2)	17.1 (16.4, 17.7)	<0.0001
Gait speed (m/s)	1.08 (1.07, 1.09)	0.80 (0.78, 0.82)	<0.0001

Abbreviations: BMI, body mass index; RASM, relative appendicular skeletal muscle mass.

^a^Analysis of covariance or logistic regression analysis.

^b^Mean (95% confidence interval) (all such values).

The crude and adjusted relationship between chili and sweet pepper consumption and the prevalence of sarcopenia was presented in Table 2. The crude ORs (95% CI) of sarcopenia across categories of chili consumption were 1.00 (reference) for almost never, 0.65 (0.49, 0.84) for ≤ 1 time/week, 0.50 (0.34, 0.71) for ≥ 2-3 times/week (*P* for trend < 0.0001). In the final multivariate logistic model, the adjusted ORs (95% CI) of sarcopenia across categories of chili consumption were 1.00 (reference) for almost never, 0.73 (0.55, 0.97) for ≤ 1 time/week, 0.60 (0.39, 0.90) for ≥ 2-3 times/week (*P* for trend < 0.01). As for sweet pepper consumption, the crude ORs (95% CI) of sarcopenia were 1.00 (reference) for almost never, 0.65 (0.51, 0.84) for ≤ 1 time/week, 0.58 (0.41, 0.80) for ≥ 2-3 times/week (*P* for trend < 0.0001) across the three categories. After adjustment of a variety of potential confounders, the adjusted ORs (95% CI) of sarcopenia across categories of chili consumption were 1.00 (reference) for almost never, 0.73 (0.56, 0.96) for ≤ 1 time/week, 0.66 (0.45, 0.95) for ≥ 2-3 times/week (*P* for trend < 0.01). Moreover, through multiple linear regressions, after multiple adjustment, standardized *β* coefficients (95% confidence interval) of chili and sweet pepper consumption for sarcopenia level were -0.059 (-0.101, -0.022), *P* < 0.01 and -0.056, (-0.095, -0.018), *P* < 0.01, separately.

**Table 2:** Adjusted relationships of frequency of chili (or sweet pepper) consumption to sarcopenia (n = 2,451).

**Logistic regression models**	**Frequency of chili consumption**			***P* for trend ^a^**
	**almost never**	**≤1 time/week**	**≥ 2-3 times/week**	
No. of subjects	1,464	646	341	-
No. of normal	1,189	562	306	-
No. of sarcopenia	275	84	35	-
Model 1 ^b^	1.00 (reference)	0.65 (0.49, 0.84) ^c^	0.50 (0.34, 0.71)	<0.0001
Model 2 ^d^	1.00 (reference)	0.75 (0.57, 0.98)	0.57 (0.38, 0.83)	<0.01
Model 3 ^e^	1.00 (reference)	0.75 (0.56, 0.99)	0.61 (0.40, 0.90)	<0.01
Model 4 ^f^	1.00 (reference)	0.73 (0.55, 0.97)	0.60 (0.39, 0.90)	<0.01
	Frequency of sweet pepper consumption			
No. of subjects	1,250	785	416	-
No. of normal	1,011	680	366	-
No. of sarcopenia	239	105	50	-
Model 1 ^b^	1.00 (reference)	0.65 (0.51, 0.84)	0.58 (0.41, 0.80)	<0.0001
Model 2 ^d^	1.00 (reference)	0.73 (0.56, 0.94)	0.67 (0.47, 0.94)	<0.01
Model 3 ^e^	1.00 (reference)	0.75 (0.58, 0.98)	0.68 (0.47, 0.97)	0.01
Model 4 ^f^	1.00 (reference)	0.73 (0.56, 0.96)	0.66 (0.45, 0.95)	<0.01

^a^Analysis by multiple logistic regression model.

^b^Model 1 was crude model.

^c^Odds ratio (95% confidence interval) (all such values).

^d^Model 2 was adjusted for age, sex, and body mass index.

^e^Model 3 was adjusted for variables in model 2 plus physical activity, smoking status, drinking status, individual history of diseases (cardiovascular disease, stroke, cancer, diabetes, hypertension and hyperlipidemia), total energy intake, depressive symptoms, household income, marital status, educational level, employment status.

^f^Model 4 was further adjusted for dietary patterns.

## DISCUSSION

In this large-scale cross-sectional study, higher consumption of chilies and sweet pepper was correlated with a lower risk of sarcopenia. Furthermore, we also found a negative correlation of chili and sweet pepper consumption and sarcopenia severity. To our knowledge, this is the first study to investigate the relationship of chili and sweet pepper consumption with sarcopenia in the older participants of population-based cohorts.

Several pathways may explain the correlation between chili consumption and sarcopenia. Capsaicin, the phytochemical responsible for the spiciness of peppers, is a highly selective agonist for TRPV1 [10]. A previous study found that TRPV1 is present in skeletal muscle [12]. When the TRPV1 channel is activated and turned on, intracellular calcium concentrations will increase [13]. A TRPV1-mediated increase in intracellular calcium concentrations activates mTOR and promotes protein synthesis and subsequent muscle hypertrophy [15]. The activation of TRPV1 by capsaicin also can increase the expression of peroxisome proliferator-activated receptor-γ coactivator-1α (PGC-1α) in skeletal muscles [18]. PGC-1α has been reported to protect skeletal muscle from atrophy by suppressing forkhead box O-3 action and atrophy-specific gene transcription [19]. Furthermore, an animal experiment pointed out that capsaicin supplementation could improve physical activities, including grip strength and endurance performance [16]. Animal experiments also found that capsaicin might be an effective agent for protecting skeletal muscle against many metabolic disorders [20]. Therefore, the consumption of chilies may have a potentially beneficial effect on sarcopenia. Further prospective studies or randomized trials are required to clarify this finding.

In the current study, we also explored the relationship between sweet pepper consumption and sarcopenia. Likewise, frequent sweet pepper consumption was significantly correlated with a lower prevalence of sarcopenia. Capsiate, extracted from sweet pepper, is a nonpungent capsaicin-related compound. Previous experiments have explored the capsiate-induced activation of TRPV1 *in vitro* and *in vivo*. Patch-clamp experiments have demonstrated that capsiate can activate TRPV1 with a similar potency to capsaicin [21]. Capsiate can also activate TRPV1 when subcutaneously injected into hindpaws of mice [21]. Additionally, animal experiments have indicated that capsiate intake enhances the twitch force-generating capacity in mice via stimulation of TRPV1 and reduction of energy consumption [22]. Oral administration of capsiate enhanced swimming endurance of mice by stimulation of vanilloid receptors [23]. Taken together, this evidence suggests that, capsiate may be a helpful candidate for sarcopenia. Further studies would be appropriate to clarify the effect of sweet pepper consumption on sarcopenia in humans.

This study has two strengths. First, this was the first study to evaluate the relationship of the frequency of chili and sweet pepper consumption with sarcopenia in a large older population, which may inform the design of future clinical trials. Second, this study has taken into account a wide set of important confounders, such as lifestyle factors, individual histories of diseases and dietary patterns.

Despite its strengths, there are several limitations to this study. First, because of the cross-sectional design, a causal relationship and an explicit mechanism of chili and sweet pepper consumption with the risk of sarcopenia could not be determined. Future clinical trials and prospective mechanism studies are required to shed further light on this topic. Second, information regarding chili and sweet pepper consumption was obtained by a self-reported questionnaire, and there is a possibility of misreporting or recall bias. Third, muscle mass was measured by bioelectrical impedance analysis. As we know, the gold standard test is considered to be dual-energy X-ray absorptiometry. However, results of muscle mass estimation using bioelectrical impedance analysis are highly correlated with that measured using dual-energy X-ray absorptiometry [24].

In conclusion, higher chili and sweet pepper consumption were significantly related to a lower risk of sarcopenia in a large-scale adult population. Our findings suggested that capsaicin and capsiate may be natural beneficial compounds for sarcopenia.

## MATERIALS AND METHODS

### Study population

The population included participants from the Tianjin Chronic Low-grade Systemic Inflammation and Health (TCLSIH) cohort study, a large prospective dynamic cohort study initiated to explore the relationships between chronic low-grade systemic inflammation and health status. Furthermore, the comprehensive geriatric assessments are involved in the TCLSIH cohort study. Detailed information on the study design of the TCLSIH has been described elsewhere [25]. The Institution Review Board of Tianjin Medical University approved the TCLSIH and informed consent was obtained from all participants.

A total of 2,698 participants have attended a health check during the research period. We excluded those who did not complete data collection on food frequency questionnaire or measurements of muscle mass, grip strength and gait speed (n = 247). Owing to these exclusions, the final cross-sectional study population comprised of 2,451 participants.

### Assessment of chili and sweet pepper consumption

The type of chilies that participants mainly consume is finger shaped chili (*dactylus M.*), while the type of sweet pepper consumed most frequently is the bell pepper (*grossum Sent.*). According to previous studies, the spiciness grade of finger shaped chili was 9, with a capsaicin concentration of 2.919-5.838 mg/g [26, 27]. Capsiate found in the sweet pepper originating from China was 1.67±2.9 μg/g [28]. However, the total amount of bioactive ingredients in chilies and sweet peppers can be affected by environmental and nutritional conditions occurring during the cultivation such as the light intensity and temperature, the position of the fruit on the plant and the age of the fruit [29]. Estimation of the frequency of spice consumed at the individual level is emerging as a useful approach for quantifying spice intake [30]. In the present study, frequency of chili and sweet pepper consumption was estimated using a validated semi-quantitative food frequency questionnaire (FFQ) for the previous month’s intake. The reproducibility and validity of the questionnaire were assessed in a random sample of 150 participants from our cohort using data from repeated measurements of the FFQ approximately 3 months apart and 4-day weighed diet records (WDRs). The Spearman correlation coefficients between FFQ and WDRs were 0.34 for chili consumption and 0.42 for sweet pepper consumption. Spearman's rank correlation coefficients between two FFQs were 0.47 for chili consumption and 0.52 for sweet pepper consumption. Detailed information on the food frequency questionnaire has been described elsewhere [31]. A common portion size of 15g and 10g for chili, 33g and 29g for sweet pepper in males and females was specified. Chili and sweet pepper consumptions were estimated based on the responses to the following questions: “How often did you consume chilies (sweet peppers) on average during the previous month?” The response options included almost never, <1 time/week, 1 time/week, 2-3 times/week, 4-6 times/week, 1 time/day, and ≥2-3 times/day. We summarized chili and sweet pepper consumption categories as: almost never, ≤1 time/week, and ≥2-3 times/week.

### Assessment of sarcopenia

Followed the diagnostic approach of the Asian Working Group for Sarcopenia (AWGS), sarcopenia was defined by low muscle mass combined with either low muscle strength or low physical performance [1]. Severe sarcopenia was identified when all three criteria of the definition were met (low muscle mass, low muscle strength and low physical performance) [32]. Direct Segmental Multi-Frequency Bioelectrical Impedance Analysis (DSM-BIA; In-Body 720; Bio space Co., Ltd, Seoul, Korea) were applied to assess appendicular skeletal muscle mass. According to AWGS, the cutoff values of relative appendicular skeletal muscle mass (RASM), defined by appendicular skeletal muscle mass/height^2^, were 7.0 kg/m^2^ in men and 5.7 kg/m^2^ in women by using Bioelectrical Impedance Analysis.

A hydraulic hand-held dynamometer (EH101; CAMRY, Guangdong, China) was performed to measure grip strength. The grip strength was measured two times during a maximal voluntary contraction for each hand, and the highest value was used in the current study. Based on criteria of AWGS, Low muscle strength was defined as grip strength < 26 kg for men and < 18kg for women.

Gait speed over a distance of 4 m was measured to assess physical performance. Participants were requested to walk 4 m at a usual pace. A stopwatch was used to record the time. Gait speed was calculated by dividing 4 meters by the time in seconds (m/s). The reference values for low physical performance was < 1 m/s, which was made an adjustment for the participants [33].

### Assessment of other variables

Anthropometric parameters, including height and body weight, were measured by trained personnel using calibrated equipment. The body mass index (BMI) was calculated as weight (in kilograms) divided by height (in meters^2^). Sociodemographic variables, including age, sex, household income, marital status, educational level and employment status, health behaviors including physical activity, smoking status and drinking status were collected from a health status questionnaire survey. The individual history of diseases (cardiovascular disease, stroke, cancer, diabetes, hypertension, and hyperlipidemia) were assessed according to the responses to relevant questions, the personal health records [34] and annual health check. Dietary intake in the last month was assessed using a validated self-administered FFQ that included 100 food items with specified serving sizes. The FFQ included seven frequency categories ranging from ‘almost never’ to ‘≥2-3 times/day’ for foods and eight frequency categories ranging from ‘almost never’ to ‘≥4 times/day’ for beverages. The mean daily consumption of nutrients and total energy intake were calculated by an ad hoc computer program developed to analyze the questionnaire and the Chinese food composition Tables were used as the nutrient database [35]. We applied factor analysis (principal-components analysis) with varimax rotation to 100 food items and beverages (g). Based on the eigenvalue of the factors, the Scree test, and the interpretability of the derived factors, three factors were retained and labeled descriptively according to the food items showing high loading (absolute value) with respect to each dietary pattern as follows: ‘‘fruits and sweets’’ dietary pattern (factor 1), ‘‘healthy’’ pattern (factor 2), and ‘‘animal foods’’ pattern (factor 3). For each dietary pattern and each subject, we computed factor scores by summing the consumption from each food item weighted by its factor loading. A higher factor score demonstrates greater conformity to the dietary pattern. Depressive symptoms were evaluated by the Chinese version of the Zung Self-Rating Depression Scale.

### Statistical analysis

All statistical analyses were performed using Statistical Analysis System 9.3 edition for Windows (SAS Institute Inc., Cary, NC, USA). Descriptive data were described as the mean (95% confidence interval, CI) for continuous variables, and as percentages for categorical variables. For baseline characteristics, analysis of covariance was used to compare the differences of continuous variables between sarcopenia status and multiple logistic regression analysis for proportional variables. Multiple logistic regression analysis was performed with chili or sweet pepper consumption in three categories as the independent variable and the prevalence of sarcopenia as the dependent variable to evaluate the correlation between chili or sweet pepper consumption and sarcopenia. Model 1 was the crude model. Model 2 was adjusted for age, sex, and BMI. For model 3, we further adjusted potentially confounders including physical activity, smoking status, drinking status, individual history of diseases (cardiovascular disease, stroke, cancer, diabetes, hypertension, and hyperlipidemia), total energy intake, depressive symptoms, household income, marital status, educational level, and employment status. For model 4, we adjusted for variables in model 3 plus dietary patterns. Odds ratios (ORs) with their corresponding 95% CIs were presented. Furthermore, we performed a multiple linear regression analysis to evaluate the relationships between chili/sweet pepper consumption and sarcopenia level based on AWGS [32], adjusting for all covariates selected for model 4. Standardized *β* coefficients were also calculated. All tests were two-tailed and Statistical significance was set as *p* < 0.05.
